# Integrated chemical and genomic analysis of lipopeptides produced by *Bacillus velezensis* CMRP4489 with antifungal activity

**DOI:** 10.1038/s41598-026-59086-6

**Published:** 2026-07-06

**Authors:** Maria Luiza A. Jesus-Nicoletto, Julia P. Baptista, Sandriele A. Noriler, Paula O. Gouveia, Alicya M. Bertoli, Daniel V. Silva, Priscila G. Camargo, Fernando Macedo, João P. Oliveira, Ulisses P. Pereira, João C. P. Mello, Claudio R. Novello, Ulisses Rocha, Admilton G. de Oliveira

**Affiliations:** 1 Department of Microbiology, State University of Londrina, Londrina, Brazil; 2 Department of Chemistry, State University of Londrina, Londrina, Brazil; 3 Postgraduate Program of Bioinformatics, Federal University of Technology of Paraná, Cornélio Procópio, Francisco Beltrão, PR Brazil; 4 Department of Preventive Veterinary Medicine, State University of Londrina, Londrina, Brazil; 5 Department of Pharmacy, State University of Maringá, Maringá, Brazil; 6 Academic Department of Chemistry and Biology, Federal University of Technology of Paraná, Francisco Beltrão, PR Brazil; 7 Department of Computational Biology and Chemistry, Helmholtz Centre for Environmental Research– UFZ GmbH, Leipzig, Germany

**Keywords:** *Sclerotinia sclerotiorum*, Biological Control, Antifungal Activity, Mass spectrometry, Biosynthetic gene clusters, *Bacillus velezensis*., Environmental biotechnology, Antifungal agents

## Abstract

Biological control by microorganisms is an increasingly interesting alternative for reducing crop losses caused by phytopathogens. The strain *Bacillus velezensis* CMRP4489 (LABIM40) stands out for its strong biotechnological potential for controlling fungal phytopathogens, and, according to in silico analyses, 12 significant gene clusters in its genome encode secondary metabolite biosynthetic pathways. The present study aimed to produce, extract, identify, and evaluate in vitro and in silico the antifungal metabolites produced by *B. velezensis* CMRP4489 with activity against *Sclerotinia sclerotiorum*, a fungus responsible for white mold. The compound with the highest activity was identified by high-resolution electrospray ionization mass spectrometry (HR-ESI-MS) analyses and nuclear magnetic resonance (NMR) spectroscopy as bacillopeptin, a molecule of the iturin family. As a result, we included comparative genomics of the iturin family and related cyclic lipopeptides and found at least three biosynthetic gene clusters (BGCs) associated with these compounds. To our knowledge, the present study is the first to report the antifungal activity of bacillopeptins against *S. sclerotiorum*.

**Supplementary Information:**

The online version contains supplementary material available at 10.1038/s41598-026-59086-6.

## Introduction

Ensuring food security for a global population projected to reach 10 billion by 2050 is one of the greatest challenges facing modern agriculture^1^. In this scenario, crop losses caused by phytopathogens pose a major threat to agricultural productivity, with annual yield reductions in globally important crops such as wheat, rice, maize, potato, and soybean estimated at 17% to 30%^2^. Among these pathogens, fungi are particularly devastating, accounting for approximately 70–80% of all plant diseases worldwide^3^. Among fungal phytopathogens, *Sclerotinia sclerotiorum*, an ascomycete responsible for causing stem rot or white mold, is among the most devastating and non-specific plant pathogens. This fungus infects more than 400 plant species worldwide, including economically important crops such as sunflower (*Helianthus annuus*), common bean (*Phaseolus vulgaris*), soybean (*Glycine max* (L.) Merrill), canola (*Brassica napus*), and ornamental plants, causing yield losses that can reach up to 100% of the crop^4,5^.

Chemical control is one of the leading methods for reducing such losses. However, its excess poses environmental and health risks, as well as the risk of pathogen resistance to the product. As a result, there is a growing need to develop alternative, innovative methods to control severe losses caused by phytopathogens in crops^6^.

One alternative gaining interest is biological control, which uses natural antagonistic microorganisms known as microbial biocontrol agents (mBCAs). Biological products based on beneficial microbial strains, such as Plant Growth-Promoting Bacteria (PGPB), represent a promising alternative as microbial biological control agents (MBCAs). PGPB are beneficial bacteria that enhance plant growth and increase tolerance to both biotic and abiotic stresses. These microorganisms can inhabit the soil as free-living organisms or colonize the rhizosphere, phyllosphere (epiphytes), and internal plant tissues (endophytes)^7,8^. Among the most widely studied PGPB are species belonging to the genus *Bacillus*. These Gram-positive soil bacteria promote plant growth and health through multiple mechanisms, including the induction of systemic resistance (ISR), competition for ecological niches and nutrients, and the production of antimicrobial compounds that suppress pathogens through antibiosis^9^. Among the species of the genus *Bacillus*, *B. velezensis* deserves mention as an alternative for biological control. These bacteria are promoters of plant growth and producers of secondary metabolites with antimicrobial activity, such as lipopeptides (surfactin, fengycin, and iturin), polyketides (macrolatin, bacillaene, and difficidin), and peptides (plantazolicin, amylocyclicin, and bacilysin)^10–12^.

Bacillopeptins are cyclic lipopeptides composed of seven amino acids (L-Asn1-D-Tyr-D-Asn2-L-Ser1-L-Glu-D-Ser2-L-Thr) and one β-amino fatty acid^13^. These lipopeptides belong to the iturine family, characterized by the conserved amino acids tyrosine and asparagine in the second and third positions of the polypeptide chain, respectively. Iturins have broad antifungal activity but limited antibacterial activity and no reported antiviral activity^14^, possibly due to their different mechanism of action. Instead of breaking the membrane or solubilizing as surfactins do, iturins form ion-conducting pores, leading to osmotic disturbance^15,16^.

Previous studies by Baptista and collaborators investigated the antifungal activity and genomic features of *Bacillus velezensis* CMRP4489, highlighting its potential as a biocontrol agent^17,18^. They identified 12 important gene clusters involved in secondary metabolite biosynthetic pathways, which may be responsible for its antifungal activity, although the metabolites underlying its broad antifungal activity have not yet been identified.

This study extracted, identified, and evaluated antifungal metabolites produced by *Bacillus velezensis* CMRP4489 using in vitro and in silico approaches against *Sclerotinia sclerotiorum*.

## Methods

### Microorganisms

*B. velezensis* CMRP4489 was initially isolated as an antagonist contaminant of a fungal culture plate, and since then, its biotechnological potential has undergone investigation^17,18^. This strain is cryopreserved at the Laboratory of Microbial Biotechnology of the State University of Londrina (LABIM/UEL). *B. velezensis* CMRP4489 is registered in the National System for the Management of Genetic Heritage and Associated Traditional Knowledge – SisGen, under registration No. A7DAEBE, and deposited in the Microbiological Collection of the Paraná Network (CMRP) of the Federal University of Paraná, Curitiba, under No. CMRP4489.

The fungal pathogen *S. sclerotiorum* used in this study was kindly provided by Professor Dr. Maria Isabel Balbi-Peña of the Laboratory of Phytopathology at UEL.

The activation of the CMRP4489 strain was performed by inoculating a plate containing Luria-Bertani agar (LB – Acumedia, USA) and incubating it at 28 °C for 24 h. To prepare the pre-inoculum, isolated colonies were suspended in saline solution (0.85% sodium chloride) and adjusted to 0.5 on the McFarland nephelometric scale (1.5 × 10^8^ CFU). A total of 30 µL of the suspension was inoculated into a 250 mL Erlenmeyer flask containing 30 mL of Luria-Bertani broth (LB – Acumedia, USA) and incubated at 28 °C for 24 h at 200 rpm (Orbital Shaker Tecnal – TE 422, Brazil).

### Production of the antifungal metabolites

To produce the antifungal metabolites, a 4 mL aliquot of the inoculum was transferred to a 1000 mL Erlenmeyer flask containing 400 mL of CM2 medium that consisted of (g/L): tryptone 12.4; glucose 20; NaCl 5; K_2_HPO_4_.3H_2_O 1.5; MnSO_4_·H_2_O 0.04; FeSO_4_.7H_2_O 1.67; MgCl_2_.6H_2_O 1.22; pH 7.1 (Patent BR 10 2020 013481 7). All components were of analytical grade; tryptone and glucose were obtained from Acumedia (USA), and the salts were purchased from Synth (Brazil). The inoculated medium was cultivated at 28 °C for 72 h at 200 rpm, then centrifuged for 10 min at 8860 × g (Hitachi CR21G Himac, Japan). The cell-free supernatant was lyophilized (Liobras, L101, Brazil).

## Extraction and purification

### Vacuum liquid chromatography

One gram of cell-free lyophilized supernatant was fractionated by vacuum liquid chromatography (VLC), with a glass column packed with silica gel 60 (0.063–0.200 mm), using five eluting systems with increasing polarity, all using analytical-grade solvents (> 99%) acquired from Synth (Brazil): 100% ethyl acetate, ethyl acetate/methanol [1:1], 100% methanol, methanol/water [1:1], and 100% water. The collected samples were concentrated in a rotary evaporator under reduced pressure (Fisatom 801, Brazil) to obtain fraction yields. The antifungal activity of each fraction was tested using disk diffusion tests, as described in the topics below.

### Flash liquid chromatography

The fraction presenting the best antifungal activity obtained after VLC was again fractionated via flash liquid chromatography (FLC) with a glass column packed with silica gel 60 (0.04–0.063 mm), using three eluting systems with increasing polarity, all using analytical-grade solvents (> 99%) acquired from Synth (Brazil): ethyl acetate/methanol [1:1], methanol/butanol [5:4], and methanol/water [1:1]. The collected samples were concentrated in a rotary evaporator under reduced pressure (Fisatom 801, Brazil) to obtain the yield of the FLC fractions. Spot-on-lawn tests monitored the antifungal activity of the fractions. The fraction’s minimum inhibitory concentration (MIC), which indicates the highest antifungal activity, was also determined. These tests were performed as described in the following topics.

### Preparative high-performance liquid chromatography

The fraction showing the most significant antifungal activity was subjected to preparative high-performance liquid chromatography (prep HPLC) on a Shimadzu LC-6AD liquid chromatograph using an RP-C18 column (5 μm, 20 × 250 mm; Shimadzu, Japan). The antifungal fraction was redissolved in acetonitrile, filtered through a 0.22 μm filter (Millipore, Billerica, MA), and 1 mL was injected into the instrument’s injector port using a Hamilton HPLC syringe. The wavelength for UV detection was 294 nm. Elution was conducted at a flow rate of 4.0 mL/min at 25 °C. The mobile phase consisted of water and acetonitrile (HPLC grade ≥ 99.9%, Honeywell, Germany), with isocratic elution at 30% acetonitrile for 60 min. The antifungal activity of each compound obtained after prep-HPLC was monitored using spot-on-lawn assays, as described in the sections below.

### Thin-layer chromatography

The chemical profiles of the obtained fractions were analyzed by thin-layer chromatography (TLC) on 0.200 mm-thick aluminum chromatography sheets (silica gel 60) using 5 µl of each fraction. Different mobile systems all using analytical-grade solvents (> 99%) acquired from Synth (Brazil) (v/v) (butanol/methanol [1:1]), (butanol/methanol [1:1]), (butanol/methanol [6:4]), (methanol/butanol [7:3]), (methanol/butanol [6:4]), and (butanol/ethanol [1:1]) were tested to discover which one was the best for visualizing the chemical profile of the fractions obtained by VLC and FLC. The TLCs were visualized under UV light at wavelengths of 365 nm and 254 nm.

Additionally, the TLCs of the active compound obtained from the prep HPLC were developed using the ninhydrin reagent (0.1 g of Ninhydrin, 0.5 mL of acetic acid 99,7%, and 100 mL of acetone 99,5%) to assess the presence of amino acids.

### Biological assays for activity monitoring

#### Disk diffusion test

The fractions obtained from VLC were evaluated for antifungal activity using disk diffusion assays. For each test, 1000 µg of each fraction diluted in 10 µL of methanol (99,8%) was applied to individual paper disks. A 5 mm-diameter plug of *S. sclerotiorum* was placed at the center of a 90-mm Petri dish containing potato dextrose agar (PDA), and the discs impregnated with the fractions were positioned at opposite edges of the dish. In all antifungal activity assays, the diameter of the inhibition halos was determined as the average of two orthogonal measurements (vertical and horizontal), both taken from the center of the halo. Each assay was performed in triplicate.

#### Spot-on-lawn test

To monitor the antifungal activity of the FLC and preparative HPLC fractions, 500 µg and 250 µg of each fraction were diluted in 10 µL of water and applied to 140 mm-diameter Petri dishes containing PDA medium. Subsequently, four plugs of *S. sclerotiorum* were placed at the edges of each plate. The plates were then incubated at 25 °C for 72 h. All assays were performed in triplicate.

#### Minimum inhibitory concentration test

The minimum inhibitory concentration (MIC) of the most active FLC fraction was determined using the method described by Simionato and collaborators^19^, in which serial dilutions of the most active FLC fraction were prepared in Petri dishes containing 10 mL of the PDA medium incorporated with different gradual concentrations of the fraction (0.7 to 50 µg/mL). Plugs measuring 5 mm in diameter containing the mycelium of *S. sclerotiorum*, obtained from a 3-day culture, were placed at the center of plates containing the solidified medium containing the compound. Mycelial growth was evaluated during a 3-day incubation at 25 °C, and the percentage of mycelial growth inhibition (MGI) was calculated according to Yahyazadeh and colleagues^20^:

1 $$\:MGI\left(\%\right)=\left[\frac{\left(C-T\right)}{C}\right]X100$$

where C (mm) is the average colony diameter in the control, and T (mm) is the average colony diameter for each treatment. The minimum inhibitory concentration indicated the lowest concentration that inhibited fungal growth.

### Statistical analysis

The diameters of the inhibition halos were subjected to statistical analysis using R (R Core Team, 2023), and the assumptions of residual independence, normality of errors, and homogeneity of variances were evaluated using the Durbin-Watson, Shapiro-Wilk, and Bartlett tests (*p* < 0.05), respectively. The data were analyzed using ANOVA, and means were compared using Tukey’s test at a 5% significance level.

### High-resolution electrospray ionization mass spectrometry analyses

The purified active fraction was analyzed in a Shimadzu Prominence UFLC system coupled to a quadrupole time-of-flight mass spectrometry (Q-TOF-MS) (Compact-Bruker Daltonics, EUA) equipped with an electrospray ionization source. A 5 µL aliquot of the sample was injected onto a Shimadzu Shim-pack XR-ODS C18 column (2.0 × 75 mm, particle size 5 μm). The mobile phases employed were water with 0.1% formic acid (LC/MS Grade ≥ 99.9%, Sigma-Aldrich, Germany) (A) and methanol with 0.1% formic acid (LC/MS Grade ≥ 99.9%, Sigma-Aldrich, Germany) (B). The following linear gradient elution was used: 60% A at 0 min, decreased to 20% A from 0 to 10 min, held at 20% A from 10 to 14 min, then increased to 60% A from 14 to 15 min. The flow rate was set at 200 µL/min. The mass spectra were recorded in ESI-positive mode; the capillary voltage was 4.5 kV, the desolvation temperature was 200 °C, and the collision energy was 8 eV.

### Nuclear magnetic resonance spectroscopy analyses

The ^1^H, COSY (COrrelation SpectroscopY), ¹H, ³C-HMBC (Heteronuclear Multiple Bond Correlation), ¹H,³C-HSQC (Heteronuclear Single Quantum Correlation), NOESY (Nuclear Overhauser Effect SpectroscopY), and DOSY (Diffusion-Ordered SpectroscopY) Nuclear Magnetic Resonance (NMR) spectra were acquired in a Bruker spectrometer model Avance III (Bruker Biospin, Rheinstetten, Germany), operating at 400.13 MHz for ^1^H and 100.13 MHz for ^13^C, at 25 °C temperature, equipped with a 5 mm multinuclear probe. Dimethylsulfoxide (DMSO-*d*_*6*_, 2.50 ppm) was used as a deuterated solvent, and tetramethylsilane (TMS, 0.00 ppm) as the internal standard. The multiplicity of protons in the ^1^H NMR spectra is reported as singlet *(s)*, doublet *(d)*, and multiplet *(m)*. Coupling constants *(J)* are reported in Hz.

### Genome mining of *B. velezensis* CMRP4489 for lipopeptides production

The genome and the secondary metabolites produced by *B. velezensis* CMRP4489 were previously described^17^. This study focused on exploring lipopeptide gene clusters, primarily those involved in the production of bacillopeptins and related compounds. This analysis was based on the study performed by Dunlap^21^ for associated iturins and lipopeptides. Here, we included one representative strain of each species in the B. subtilis group, as used by Dunlap and collaborators^21^(Supplementary 1, Table S1). The characterized gene clusters for lipopeptides in the MIBiG database were also included (Supplementary 1 Table S1). The biosynthetic gene cluster (BGC) from *B. velezensis* CMRP4489 was predicted by antiSMASH 7.0^22^, and the BGCs related to lipopeptide production were manually curated. The core enzymes associated with these BGCs, belonging to the non-ribosomal peptide class, were curated using BGCtoolkit v0.3.7. The adenylation (A) domains were extracted as described by Noriler and collaborators^23^. The A domains were used to construct phylogenetic analyses in PhyloSuite^24^, aligned with MAFFT v. 7, and a maximum-likelihood phylogenetic tree was built using IQ-TREE v. 1.6.12^25^. iTOL was used to visualize the tree^26^. Similarity analyses of the selected BGCs were made using Clinker^27^.

### Scanning electron microscopy (SEM)

To evaluate the effect of the active compounds, plugs of *S. sclerotiorum* exposed to 250 ug of the active compounds were subjected to SEM observations. The samples were fixed in 2.5% glutaraldehyde (25%), 2% paraformaldehyde (> 95%) in 0.1 M sodium cacodylate (≥ 98%) buffer, pH 7.2, for 12 h. The samples were then washed with 0.1 M sodium cacodylate buffer (pH 7.2) for 10 min, and the wash was repeated 3 times. The fixed material was dehydrated in a gradient of ethanol concentration. The samples were then dried using the critical point method with CO_2_ in a BALTEC CPD 030 critical point dryer, coated with gold (BALTEC SDC 050 Sputter Coater), and observed in an FEI Quanta 200 SEM operating at 30 kV^19^.

## Results

### Extraction and purification of the antifungal compound

Five fractions (F1-F5) were obtained after the VLC of the cell free supernatant. To determine the chemical profiles of each fraction, several TLCs were performed using different mobile phases. According to the results, a water-methanol-butanol (2:4:4, v/v) solution was the optimal eluent for separating the components of each fraction. The antifungal activity of the fractions was monitored using a disk diffusion test.

The bioactive fraction (F2) was eluted again with methanol/butanol/water [5:4:1] in FLC, and the antifungal compound (fraction F6F) was extracted (15.75 mg). All fractions were monitored by TLC using the eluent butanol/methanol/water [6:3:1, v/v], the best eluent tested. In TLC, the fraction F6F showed bands with R_*f*_ of 0.32 and 0.47. The F6F fraction was subjected to prep HPLC, yielding four compounds: F6F.1, F6F.2, F6F.3, and F6F.4.

### Biological assays for activity monitoring

Of the fractions resulting from the VLC, only F2 showed activity against S. sclerotiorum in the disk diffusion test, with 16 ± 0.8 mm inhibition halos, while the lyophilized cell-free supernatant (crude extract) showed 15.5 ± 0.8 mm inhibition halos.

Spot-on-lawn tests were conducted to determine which fraction was obtained after FLC showed antifungal activity (Supplementary Fig. S1). Among the nine fractions obtained (F1F-F9F), F6F, F7F, and F8F exhibited antifungal activity against *S. sclerotiorum*, with inhibition halos measuring 26.25 ± 1.8, 23 ± 1.0, and 15 ± 0.5 mm, respectively. F6F showed the most significant activity (*p* < 0.05), with inhibition halos of 9 ± 1.0 mm at the same concentration as F2 (500 µg).

**Fig. 1:**
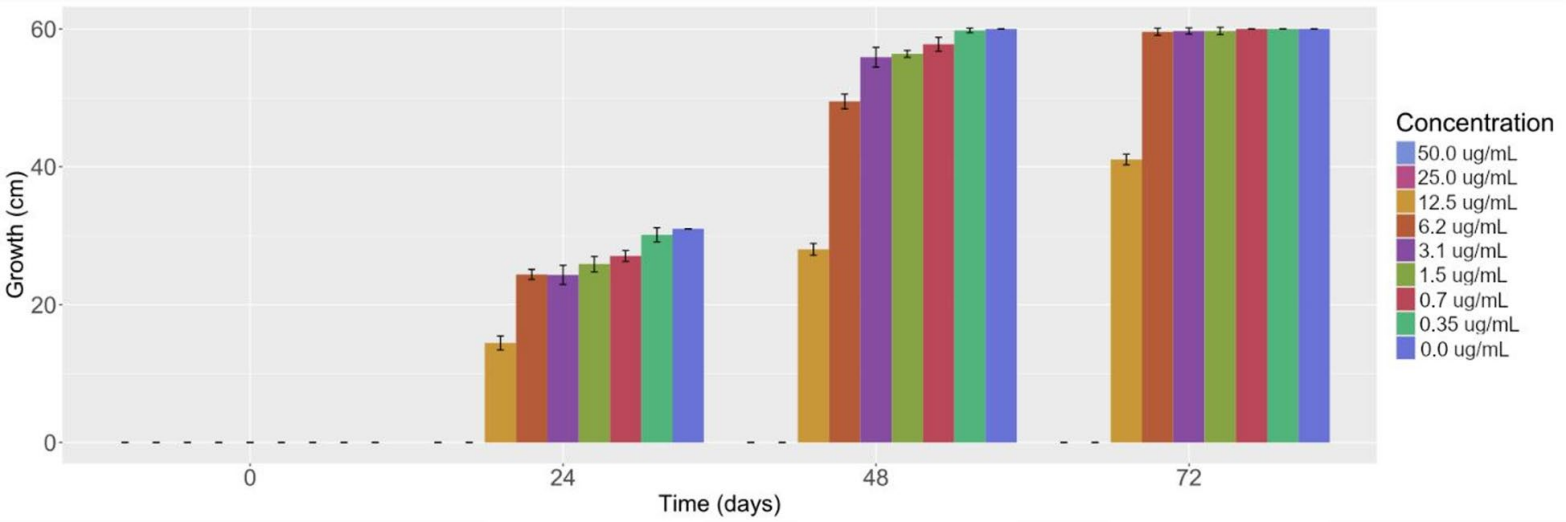
Effect of different concentrations of fraction F6F on the mycelial growth of *Sclerotinia sclerotiorum* over time. Fungal growth (cm) was measured at 0, 24, 48, and 72 h in PDA medium supplemented with F6F at concentrations of 50, 25, 12.5, 6.2, 3.1, 1.5, 0.7, 0.35, and 0 µg/mL. Within each time point, bars are arranged from left to right in decreasing concentration (50 to 0 µg/mL), as indicated in the color legend. Data represent the mean ± standard deviation of three independent experiments, and error bars indicate standard deviation.

We conducted MIC tests to determine the lowest MIC of F6F required to inhibit *S. sclerotiorum*. F6F was selected to continue the purification process because it exhibited the best antifungal activity (Fig. 1). The concentration of F6F that completely inhibited *S. sclerotiorum* growth was 25 µg/mL; when reduced by half, mycelial growth also decreased by half. Concentrations below 12.5 µg/mL showed lower inhibition, with growth remarkably similar to the control, as shown in Fig. 1.

**Fig. 2:**
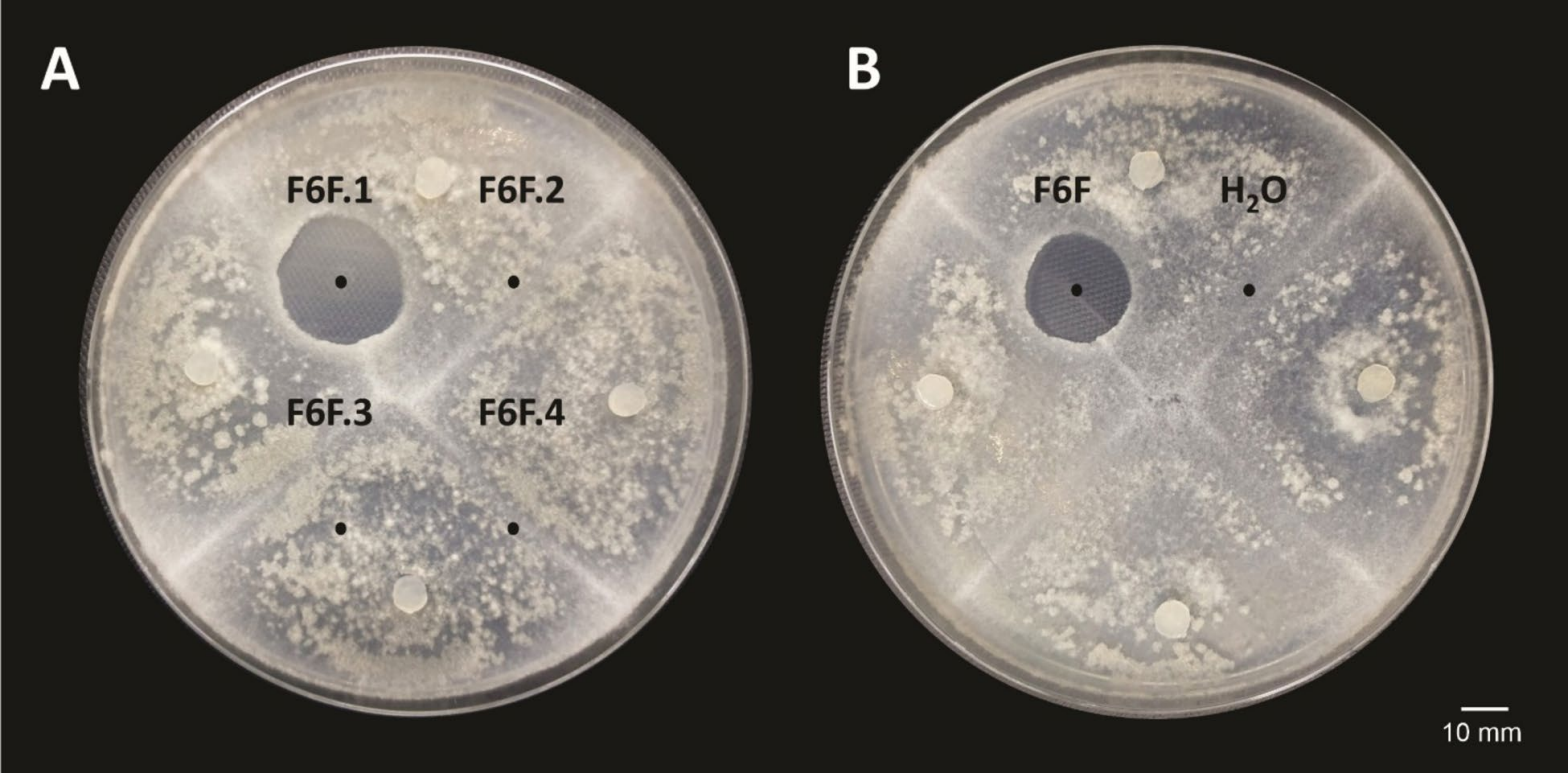
Spot-on-Lawn assay of the fractions obtained by prep HPLC using F6F. A - Inhibition halos were produced in the Spot-on-Lawn assay using 250 µg of the fractions obtained by prep HPLC in a 150 × 15 mm plate. B – Inhibition halo of the F6F fraction using 250 µg, and the control using water to elute the fractions in a 90 × 15 mm plate. Only F6F.1 produced inhibition halos against *S. sclerotiorum*, aside from F6F.

Among the four compounds obtained in prep HPLC, only F6F.1 produced inhibition halos, measuring 27.5 ± 2.0 mm with 250 µg in the spot-on-lawn test. Meanwhile, F6F presented halos of 18.1 ± 0.4 mm at the same concentration (Fig. 2).

### Active compound identification

The ninhydrin test of F6F.1 in TLC was positive for amino acids (a single band with R_*f*_=0.325). In the positive UFLC-HR-ESI-MS spectra, two peaks were observed at *m/z* 1021.5379 [M + H]^+^ (Supplementary Fig. S2) and *m/z* 1035.5620 [M + H]^+^ (Supplementary Fig. S3), consistent with the molecular formulas C_46_H_72_N_10_O_16_ (calc.1021,5207) and C_47_H_74_N_10_O_16_ (calc. 1035.5363). These peaks suggest the presence of two compounds in the mixture, denoted as **A** and **B**. The additional peaks at m/z 1043.4992 further supported the presence of two compounds: [M + Na]+ (A) and [M + Na]+ (B) at m/z 1057.5418.

Compounds **A** and **B** were characterized with bacillopeptin A and bacillopeptin B by ^1^H NMR (Table 1), DOSY, COSY, HMBC, HSQC, and NOESY (Supplementary Fig. S4 to Fig. S10) compared with reported data (13–17,28). Based on the obtained spectra, fraction F6F.1 is composed mainly of bacillopeptins A and B, with only minor interfering compounds present at low concentrations.

**Table 1:** ^1^H and ^13^C NMR data of **1** and **2** in DMSO-d_6_ (*δ* in ppm, *J* in Hz)^a^ compared with the literature data for bacillopeptin A and B^b^. From the NMR spectra, it was possible to identify seven amino acid residues, in addition to an aliphatic carbon chain in compounds 1 and 2, similar to bacillopeptin A and B, respectively.

Amino acids	Position	1 and 2		Bacillopeptin A	
		δ_C_	δ_H_	δ_C_	δ_H_
Asn	1	50.2	4.43 dd (6.5–13.0)	50.1	4.45 dd (7.0–13.2)
	2	37.3	2.30 m	36.7	2.28 m
	3	171.9		171.6	
	4	172.0		171.8	
	1-NH		8.01 brd		8.02 brd
	3-NH_2_		6.92		7.41
Tyr	5	55.2	4.25 m	55.7	4.22
	6	35.9	2.91 m	35.6	2.69; 2.94
	7	128.2		128.1	
	8/12	130.5	7.00 d (8.31 Hz)	130.1	7.00 (d, 8.4 Hz)
	9/11	115.4	6.64 d (8.31 Hz)	115.2	6.63 (d, 8.4 Hz)
	10	156.3		155.9	
	13	172,3		171.4	
	5-NH		8.29 m		8.29 (d, 7.1 Hz)
	10-OH		10.87		9.20
Asn	14	50.6	4.47 dd (6.5–14.0)	50.8	4.47
	15	36.8	2.57 m	36.8	2.50; 2.56
	16	171.8		171.5	
	17	171.6		171.3	
	14-NH		8.35		8.04
	16-NH_2_		6.84; 7.42		6.95; 7.31
Ser	18	55.2	4.29 m	55.2	4.23
	19	61.5	3.62 m	61.2	3.57
	20	172.2		169.6	
	18-NH		7.72		7.56
Glu	21	54.1	4.12	53.0	4.18
	22	27.5	1.88/1.90	25.4	1.88/1.94
	23	32.6	2.20 m	31.4	2.22
	24	172.0		174.3	
	25	171.8		172.4	
	21-NH		8.30		8.00
Ser	26	55.4	4.31 dd (6.0–12.0)	55.3	4.33
	27	61.4	3.66 m	61.4	3.60
	28	171.8		170.3	
	26-NH		8.10		8.13
Thr	29	59.3	4.04	58.8	4.05
	30	66.4	4.10	66.0	4.06
	31	19.8	1.01 d (6.10)	20.1	1.01 d (6.12)
	32	170.0		170.7	
	29-NH		7.75		7.69
RA	Position	**1**		Bacillopeptin A	
		δ_C_	δ_H_	δ_C_	δ_H_
	33			171.2	
	34	40.7	2.28	40.9	2.28
	35	46.6	3.93 m	46.3	3.98
	36	34.0	1.37	34.0	1.34/1.38
	37	25.4	1.10/1.19	25.4	1.12/1.21
	38–44	29.4	1.19	28.8–29.2	1.21
	45	27.8	1.19	22.0	1.26
	46	14.3	0.80	13.9	0.85
	35-NH		7.42		7.28
RB	Position	**2**		Bacillopeptin B	
		δ_C_	δ_H_	δ_C_	δ_H_
	33			171.2	
	34		2.28	40.9	2.28
	35	46.6	3.93 m	46.3	3.98
	36	34.0	1.37	34.0	1.34/1.38
	37	25.4	1.10/1.19	25.4	1.12/1.21
	38–43	29.4	1.19	28.8–29.2	1.21
	44	38.7	1.12	38.4	1.14
	45	27.8	1.47	27.3	1.49
	46	22.9	0.84	22.4	0.84
	47	22.9	0.82	22.4	0.82
	35-NH		7.42		7.28

^a^ All assignments were made by extensive analyses of 1D and 2D NMR (COSY, NOESY, HSQC, and HMBC). ^b^ Data extracted from Kajimura and collaborators^13^.

Using DOSY (Supplementary Fig. S4), it was possible to identify the set of NMR peaks in F6F.1 corresponding to metabolites within the desired molecular weight range, as indicated by the HRMS peaks. Pseudo-2D DOSY plots showed well-resolved signals with diffusion coefficients between 0.8 × 10^− 10^ m^2^ s^− 1^ and 0.9 × 10^− 10^ m^2^ s^− 1^. Protons that have a diffusion coefficient between 0.8.10^− 10^ m^2^ s^− 1^ and 0.9.10^− 10^ m^2^ s^− 1^ are shown in Table 1.

Bacillopeptin A and B have very similar molecular structures. The only structural difference was the presence of an additional methyl group at C-45 in the alkyl chain of bacillopeptin B. As a result, most of the signals observed in the NMR spectra of compounds A and B overlap and have been assigned to both molecules.

The ^1^H NMR spectrum (Supplementary Fig. S5) presented mainly eleven signals (δ 6.84–8.29) of N-bonded protons (–NH– or –NH_2_) and eight *α*-amide protons (δ 4.49–3.74), consistent with a cyclic peptide composed of seven amino acid residues. The signals at δ 6.84, 6.92, and 7.42 ppm corresponded to the terminal amide protons (-NH_2_) of the two asparagine residues. The alkyl chains were characterized by signals related to methylene (δ 1.10 to 1.37) and methyl groups (δ 0.80 to 0.84). Also, signals at δ 6.64 (2-H, J = 8.31 Hz) and 7.00 (2-H, J = 8.31 Hz) showed the presence of the p-hydroxyphenyl group of the tyrosine residue.

**Fig. 3:**
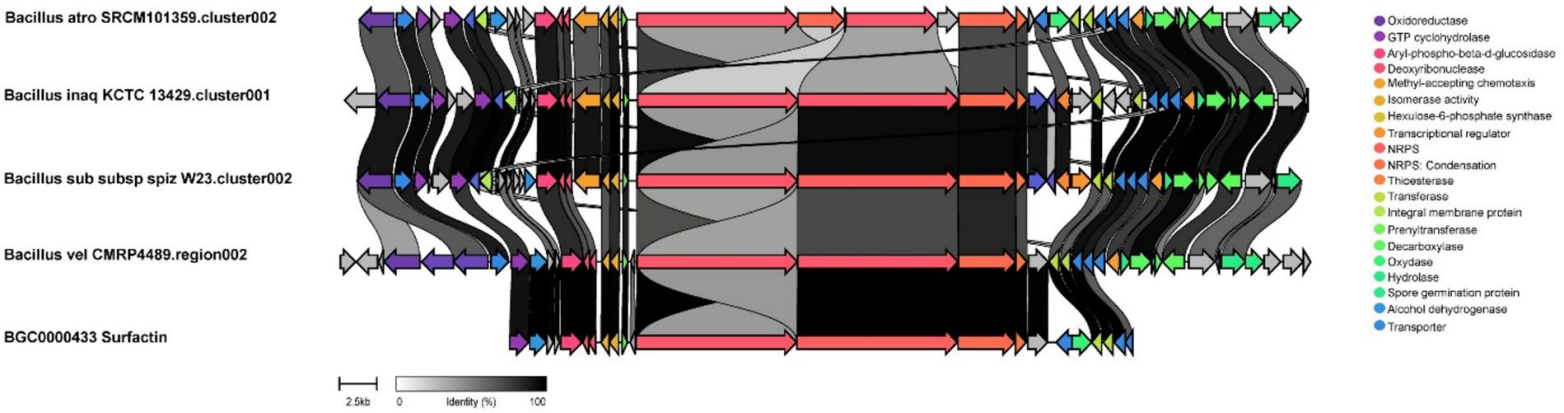
Synteny of genomic loci from the Bacillus velezensis CMRP4489 region 2, the BGC from Bacillus spp. from Dunlap et al. (2019), and BCG sequences deposited in the MIBiG database. Homology analyses were performed using the Clinker Software.

Eight spin systems observed in COSY (Supplementary Fig. S6) together with correlations present in HSQC (Supplementary Fig. S7) spectra helped identify the amino acid residues for compounds **A** and **B**: two asparagine (Asn), two serine (Ser), one glutamine (Gln), one threonine (Thr), and one tyrosine (Tyr). The carbonyl groups were assigned based on their correlations observed in the HMBC spectrum (Supplementary Figs. S8 and S9) and correlations with other quaternary carbons. Correlations observed in the NOESY spectrum (Supplementary Fig. S10) are shown in Fig. 3.

**Fig. 4:**
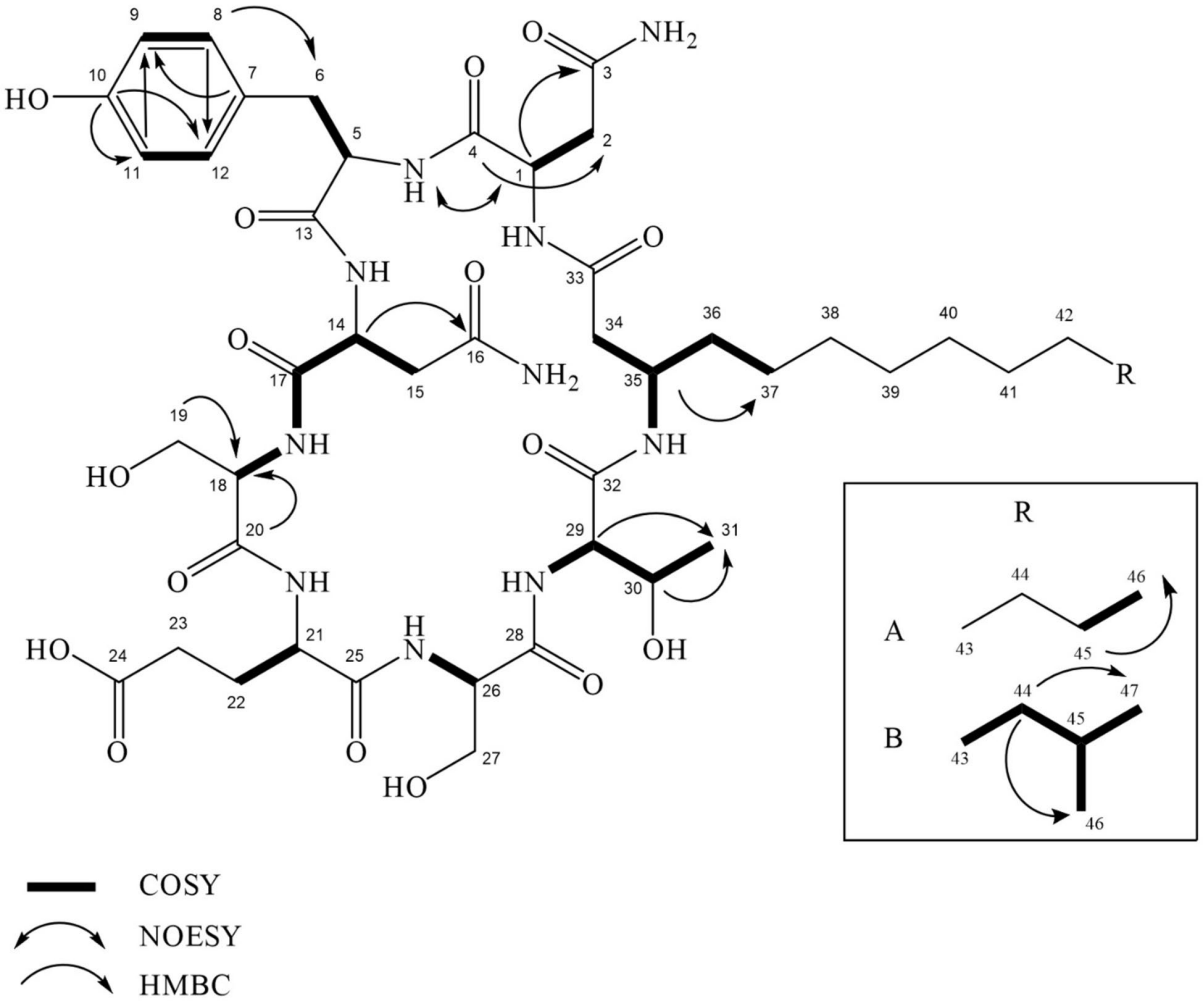
Chemical structure of Bacillopeptins produced by *B. velezensis* CMRP4489 and the correlations found in the 2D NMR spectra. The R group indicates the structural differences that differentiate bacillopeptin A from bacillopeptin B.

The n-alkyl chain, characteristic of bacillopeptin A, was proposed based on correlations observed in the COSY spectrum between the terminal methyl group at δ 0.80 ppm and the methylene groups at δ 1.19 ppm in compound **A**. In the same way, branching in the terminal portion of the alkyl chain for compound **B**, characteristic of bacillopetin B, was proposed based on correlations observed between signals for two methyl groups at δ 0.82 and 0.84 ppm and methylene groups at δ 1.47 and 1.18 ppm, respectively. These observations, combined with HMBC, HSQC, and HRMS data, compared with the literature (13, 28–33), allowed us to identify compounds A and B as bacillopeptin A and bacillopeptin B, respectively. The molecular structure and the correlations observed in the 2D spectra are shown in Fig. 4.

### Genome mining of *B. velezensis* CMRP4489 for lipopeptides production

Genome mining using antiSMASH identified three regions harboring gene clusters for lipopeptide production: regions 2, 7, and 11 are associated with the biosynthesis of surfactin, fengycin, and bacillibactin, respectively. The phylogenetic analysis grouped *B. velezensis* CMRP4489 with *Bacillus* spp. used as reference (Supplementary Fig. S11). Based on the phylogeny of the core enzymes, we found that Region 2 groups with BGC0000433 (surfactin) from *Bacillus inaquosorum* strain KCTC 13,429, *B. subtilis* subsp. *spizizenii* strain W23, and *B. atrophaeus* strain SRCM101359 (Supplementary Fig. S11). Region 7 includes the characterized BGCs from MIBiG—BGC0001095 (fengycin), BGC0001090 (bacillomycin D), BGC0001098 (iturin), and BGC0001103 (mycosubtilin)—as well as all *Bacillus* spp. strains analyzed in this study, except for *B. halotolerans* strain III-1(Supplementary Fig. S11).

Region 11 harbors BGC0000309 (bacillibactin), along with clusters from *B. inaquosorum* strain KCTC 13,429, *B. subtilis* subsp. *spizizenii* strain W23, and *B. atrophaeus* strain SRCM101359.

**Fig. 5:**
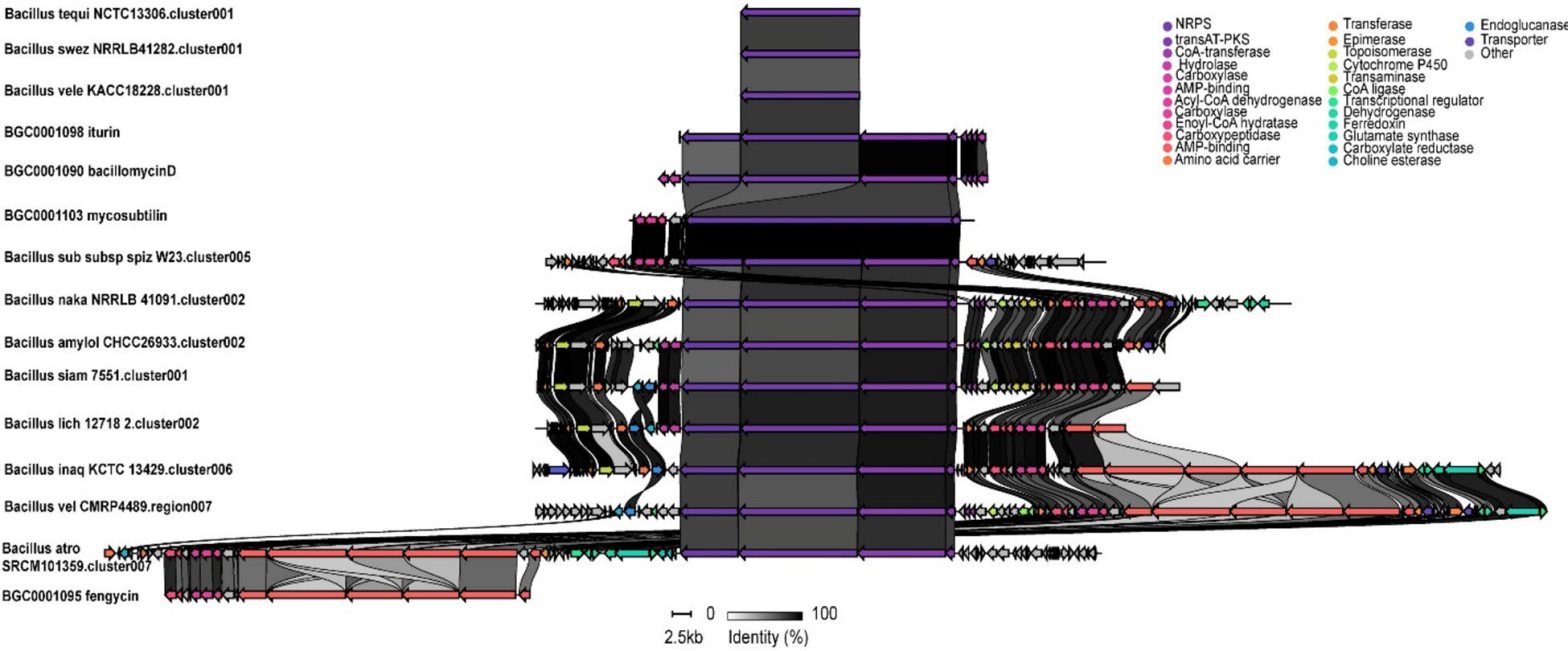
Synteny of genomic loci from the Bacillus velezensis CMRP4489 region 7, the BGC from Bacillus spp. from Dunlap and colleagues (21), and BCG sequences deposited in the MIBiG database. Homology analyses were performed using the Clinker Software.

**Fig. 6:**
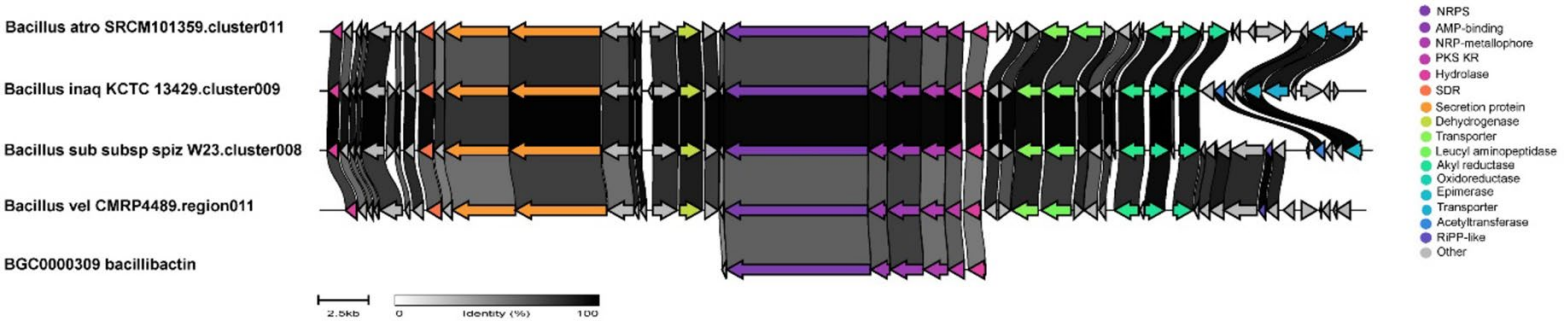
Synteny of genomic loci from the Bacillus velezensis CMRP4489 region 11, the BGC from Bacillus spp. from Dunlap and colleagues (21), and BCG sequences deposited in the MIBiG database. Homology analyses were performed using the Clinker Software. Unpredicted proteins are named as others.

**Fig. 7:**
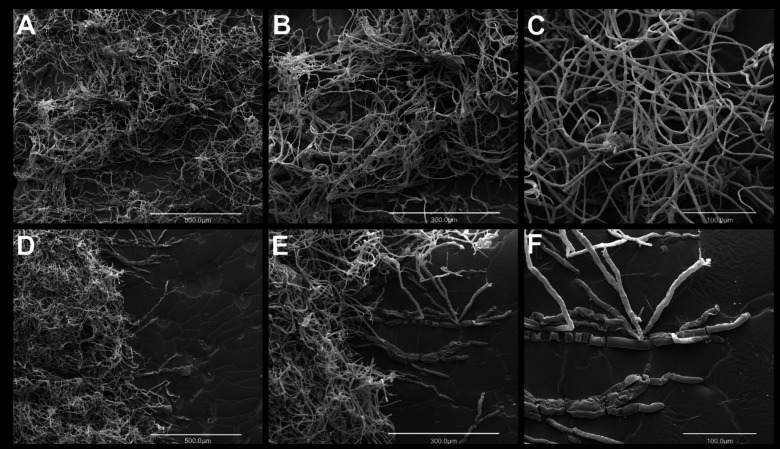
Scanning electron microscopy images of the antifungal effect of bacillopeptins against *S. sclerotiorum*. **(A-C)** Control (not treated with bacillopeptins): a large number of hyphae, demonstrating typical structure and a smooth surface. **(D-F)** Agar interface containing bacillopeptins and *S. sclerotiorum;* hypha with a rough aspect and unusual protuberances. Magnifications: 200× (A, D), 400× (B, E), and 800× (C, F).

To further investigate the similarity between the core enzymes and the complete biosynthetic gene clusters, we performed a similarity analysis (Figs. 5, 6 and 7). In Fig. 3, the surfactin cluster shows high similarity with *B. velezensis* CMRP4489 Region 2 and the corresponding reference strains, indicating that the entire cluster is conserved. The clusters responsible for iturin, bacillomycin D, mycosubtilin, and fengycin biosynthesis appear closely related to *B. velezensis* CMRP4489 Region 7, as well as to the other *Bacillus* spp. used as references for the presence of iturins and structurally related lipopeptides. However, fengycin biosynthesis appears to be derived from a distinct gene cluster organization (Fig. 5).

Our results demonstrate that *B. velezensis* CMRP4489 shares core and tailoring enzymes with *B. inaquosorum* strain KCTC 13,429 and *B. atrophaeus* strain SRCM101359 (Fig. 5). Nonetheless, some tailoring enzymes appear to be unique to the CMRP4489 strain, and further studies are required to elucidate their biosynthetic roles in lipopeptide production.

As mentioned, Region 11 is associated with bacillibactin biosynthesis. The gene cluster in this region is closely related to the bacillibactin BGCs found in the other *Bacillus* spp. analyzed, although some tailoring enzymes remain uncharacterized (Fig. 6).

### Scanning electron microscopy (SEM)

The effect of bacillopeptins on *S. sclerotiorum* was pronounced, causing significant distortion and deformation of the hyphae. In the untreated control, the mycelium exhibited the typical characteristics of *S. sclerotiorum*, with a well-organized network structure and smooth hyphal surfaces. In contrast, exposure to bacillopeptins induced noticeable morphological alterations, including a roughened appearance and abnormal protuberances, indicating that bacillopeptins effectively inhibited *S. sclerotiorum* growth (Fig. 7).

## Discussion

In previous studies, *B. velezensis* CMRP4489 showed great potential as a biocontrol agent against phytopathogens^17^. In vitro assays showed that its metabolites exhibited strong antifungal activity against various fungal phytopathogens, including *S. sclerotiorum*, *Rhizoctonia solani*, *Botrytis cinerea*, and *Macrophomina phaseolina*. Furthermore, the metabolites showed broad stability at different pH, temperature, and light ranges. In *silico* studies showed that *B. velezensis* CMRP4489 harbors gene clusters encoding secondary metabolites with antifungal activity, such as polyketide antibiotics, thiopeptides, bacteriocins, lipopeptides, and terpene^17,18^.

The results obtained in this study, through spectrometric and spectroscopic analysis, corroborate those reported by Ma and collaborators^28^ in their study on the characterization of a new lipopeptide antibiotic belonging to the bacillopeptin family produced by *B. velezensis* SH-B7. Our results also corroborate the NMR analyses of the bacillopeptin family by Kajimura and collaborators^13^, who determined the configuration of each amino acid by chiral HPLC.

Analysis of the NMR spectra demonstrated that fraction F6F.1 is predominantly composed of bacillopeptins A and B, which are associated with the observed antifungal activity. However, a limitation of this study was the inability to isolate these molecules, which prevented evaluation of their biological activity. This limitation is due to the low yield at the end of the purification process, which did not yield sufficient amounts of each compound for independent assays. Ongoing studies aim to optimize bacillopeptin production by *B. velezensis* CMRP4489 and to scale up the extraction process to enable both the individual separation of these compounds and the future development of a biochemical product for agricultural applications containing these molecules.

Bacillopeptins are lipopeptides of the iturin family with solid antifungal activity but restricted antibacterial activity^14^. Bacillopeptin A and B are similar, differing only in the terminal portion of the β-amino fatty acid chain. Bacillopeptin A has no branches on the β-amino fatty acid chain and has a single terminal methyl group. In contrast, bacillopeptin B has a branch at C45, thus containing two terminal methyl groups^13^.

A study by Volpon and collaborators^29^ demonstrated that the antifungal activity of this family depends on strong interactions with ergosterol, which is more abundant in fungal membranes than the cholesterol found in other organisms’ membranes. The mechanism of action of bacillopeptins involves penetration of the cytoplasmic membrane via their hydrophobic tails, followed by self-aggregation to form a pore that causes the extravasation of K+ ions and other vital constituents, leading to cell death^14,30^.

These lipopeptides were discovered by Kajimura and collaborators^13^, who purified them from the supernatant of *Bacillus subtilis* FR-2, an isolate found in the rhizosphere of garlic. Purification was performed by liquid-liquid extraction with butanol, followed by column chromatography on silica gel using methanol and chloroform as eluent systems. Afterward, the active fractions were purified using a Sephadex LH-20 column, followed by preparative reverse-phase HPLC using water and acetonitrile as solvents. The authors obtained three compounds: bacillopeptin A, B, and C. Since then, several reports have described the isolation of bacillopeptins produced by distinct Bacillus species^28,31–34^.

In the same year that Kajimura and colleagues reported the discovery of bacillopeptins, Eshita and Roberto^35^ isolated a molecule produced by *B. subtilis* with the same amino acid sequence as the bacillopeptins, which they named bacillomycin Lc. Bacillomycin Lc is similar to bacillopeptins; the only difference is the presence of L-Asp1 and L-Gln5 in bacillomycin Lc, instead of L-Asn1 and L-Glu5 in bacillopeptins^13,14,29^. However, Volpon and colleagues^36^ reported the synthesis of a bacillomycin Lc analog. After a thorough analysis of the NMR spectra of bacillomycin L, they discovered that the amino acid sequence of this lipopeptide was incorrect. Instead of L-Asp1 and L-Gln5, bacillomycin L has L-Asn1 and L-Glu5, which are identical to those in bacillomycin Lc or bacillopeptin.

The antifungal activity of bacillopeptins was reported against *Fusarium oxysporum*, *Aspergillus niger*, *Aspergillus oryzae*, and *Penicillium thomii*, among others^13,28,36^. However, the present study is the first to reveal the antifungal activity of bacillopeptin against *S. sclerotiorum*, the fungus responsible for white mold. This disease affects more than 400 plant species worldwide, causing losses of up to 100% of the harvest, impairing seed quality, and reducing protein and oil content^5,37^. Bacillopeptins can be an alternative for controlling *S. sclerotiorum* and reducing losses caused by this fungus. This evidence reinforces the high potential of *B. velezensis* CMRP4489 as a biological control agent.

Regarding genomic mining for lipopeptide BGCs, we identified several BGCs in the literature that are associated with the *B. velezensis* CMRP4489 strain, highlighting the strain’s capacity to produce lipopeptide metabolites. Based on the compound configuration and BGC analyses, region 7 harbors the BGC for the bacillopeptins described here, as this gene cluster shows close relationships to the lipopeptides of the iturin group, such as bacillomycin D, which share very similar molecular structures. Also, this region harbors two complexes of core enzymes that produce distinct lipopeptides: one associated with iturin, bacillomycin D, and mycosubtilin, and another associated with the fengycin cluster. Interestingly, a previous study has shown that iturinic lipopeptides are unique to each specimen^21^. However, we detected at least three BGCs in the genome of *B. velezensis* CMRP4489, highlighting this strain’s potential for secondary metabolite production. Moreover, some tailoring enzymes appear to be restricted in *B. velezensis* CMRP4489 in this region, and to demonstrate the activity of fengicyn or other compounds related to these BGCs, additional studies, including heterologous expression, should be performed. Regions 2 and 11 appear to be well conserved among the reference strains and the corresponding characterized BGCs from MIBiG, surfactin, and bacillibactin, respectively. The surfactin BGC, previously described alongside fengycin as contributing to antifungal activity, has been shown to act synergistically, enhancing the antimicrobial potential of the evaluated *Bacillus* strains^38^. Therefore, these BGCs may also contribute to the antimicrobial activity observed for *B. velezensis* CMRP4489.

Bacillopeptins constitute only one class of bioactive compounds produced by *B. velezensis* CMRP4489, suggesting that this strain may synthesize additional biologically active metabolites. Thus, this study highlights the significant potential of *B. velezensis* CMRP4489 as a source of novel bioactive molecules beyond bacillopeptins, reinforcing its promise as a biological control agent. Our results indicate that the bacillopeptins A and B identified in this work should be systematically evaluated in greenhouse assays and subsequently under field conditions to assess their effectiveness against *S. sclerotiorum* in agricultural systems. Ongoing studies aim to optimize bacillopeptin production by *B. velezensis* CMRP4489 and to scale up the extraction process, enabling the development of a biochemical product for agricultural application containing these molecules.

## Supplementary Information

Below is the link to the electronic supplementary material.

Supplementary Material 1

## Data Availability

The sequence data of B. velezensis CMRP4489 analyzed during the current study are deposited in NCBI under Reference Sequence: NZ_CP023748.1. The sequence data used from Minimum Information about a Biosynthetic Gene Cluster (MIBiG) are available under the numbers: BGC0001098, BGC0001090, BGC0001103, BGC0001095, BGC0000309, and BGC0000433. B. velezensis CMRP4489 is registered in the National System for the Management of Genetic Heritage and Associated Traditional Knowledge – SisGen, under registration No. A7DAEBE, and deposited in the Microbiological Collection of the Paraná Network (CMRP) of the Federal University of Paraná, Curitiba, under No. CMRP4489. All other data generated or analyzed during this study are included in this published article (and its Supplementary Information files).
