# Supplementary material for: Integrated chemical and genomic analysis of lipopeptides produced by Bacillus velezensis CMRP4489 with antifungal activity
Source: Sci Rep. 2026 Jul 6;16:20770. doi: 10.1038/s41598-026-59086-6 (PMC13338125; doi:10.1038/s41598-026-59086-6)
Supplement: Supplementary file 1 — Supplementary Material 1 [file 41598_2026_59086_MOESM1_ESM.pdf]

SUPPLEMENTARY MATERIAL:

Integrated chemical and genomic analysis of lipopeptides produced by *Bacillus velezensis* CMRP4489 with antifungal activity

Maria Luiza A. Jesus-Nicoletto[a], Julia P. Baptista[a], Sandriele A. Noriler[a], Paula O. Gouveia[a], Alicya M. Bertoli[a], Daniel V. Silva[a], Priscila G. Camargo[b], Fernando C. Macedo Jr[b], João P. Oliveira[c], Ulisses P. Pereira[d], João C. P. Mello[e], Claudio R. Novello[f], Ulisses Rocha[g]*, Admilton G. Oliveira[a]*

[a]Department of Microbiology, State University of Londrina, Londrina, Brazil

[b]Department of Chemistry, State University of Londrina, Londrina, Brazil

[c]Postgraduate Program of Bioinformatics, Federal University of Technology of Paraná, Cornélio Procópio, PR, Brazil

[d]Department of Preventive Veterinary Medicine, State University of Londrina, Londrina, Brazil

[e]Department of Pharmacy, State University of Maringá, Maringá, Brazil

[f]Academic Department of Chemistry and Biology, Federal University of Technology of Paraná, Francisco Beltrão-PR, Brazil

[g]Department of Applied Microbial Ecology, Helmholtz Centre for Environmental Research–UFZ GmbH, Leipzig, Germany

*Address correspondence to Ulisses Rocha, ulisses.rocha@ufz.de; Admilton Gonçalves de Oliveira, admilton@uel.br

Table S1. Strains of each species from the B. subtilis group used in this study, their accession numbers (Sequence Read Archive), Minimum Information about a Biosynthetic Gene cluster (MIBiG), and references.

Strain	Accession number	MIBiG	Reference
Bacillus amyloliquefaciens CHCC26933	GCA_003382375	N.A.[a]	https://doi.org/10.1128/AEM.01108-18
Bacillus atrophaeus SRCM101359	NZ_CP021500.1	N.A.	https://doi.org/10.3389/fmicb.2023.1287921
Bacillus licheniformis 12718_2	MIZD01000002.1	N.A.	https://doi.org/10.1128/genomea.00971-15
Bacillus halotolerans III-1	NZ_MBQV01000011.1	N.A.	https://doi.org/10.3389/fmicb.2019.01794
Bacillus nakamurai NRRL B-41091	LSAZ01000041.1	N.A.	https://doi.org/10.1099/ijsem.0.001135
Bacillus siamensis 7551	NPCI01000001.1	N.A.	https://doi.org/10.1016/j.fsigen.2017.10.001
Bacillus inaquosorum KCTC 13429	CP029465.1	N.A.	https://doi.org/10.1016/j.syapm.2013.09.006
Bacillus subtilis subsp. *spizizenii* W23	CP002183.1	N.A.	https://doi.org/10.1099/mic.0.048520-0
Bacillus swezeyi NRRL B-41282	MTJL01000042.1	N.A.	https://doi.org/10.1099/ijsem.0.002007
Bacillus tequilensis NCTC13306	UAQB01000027.1	N.A.	https://doi.org/10.1099/ijs.0.63946-0
Bacillus velezensis KACC 18228	001461835	N.A.	https://doi.org/10.1099/ijsem.0.000858
Bacillus subtilis subsp. *subtilis* str. 168	AL009126.3	BGC0000309	https://doi.org/10.1038/36786
Bacillus subtilis RB14	AB050629.1	BGC0001098	https://doi.org/10.1128/jb.183.21.6265-6273.2001
Bacillus subtilis subsp. *spizizenii* ATCC 6633	AF184956.1	BGC0001103	https://doi.org/10.1073/pnas.96.23.13294
Bacillus velezensis FZB42	AJ575642.1	BGC0000433	https://doi.org/10.1128/jb.186.4.1084-1096.2004
Bacillus velezensis FZB42	CP000560.1	BGC0001090	https://doi.org/10.1128/jb.186.4.1084-1096.2004
Bacillus velezensis FZB42	CP000560.1	BGC0001095	https://doi.org/10.1038/nbt1325

[a] N.A., Not applicable.

Figure S1

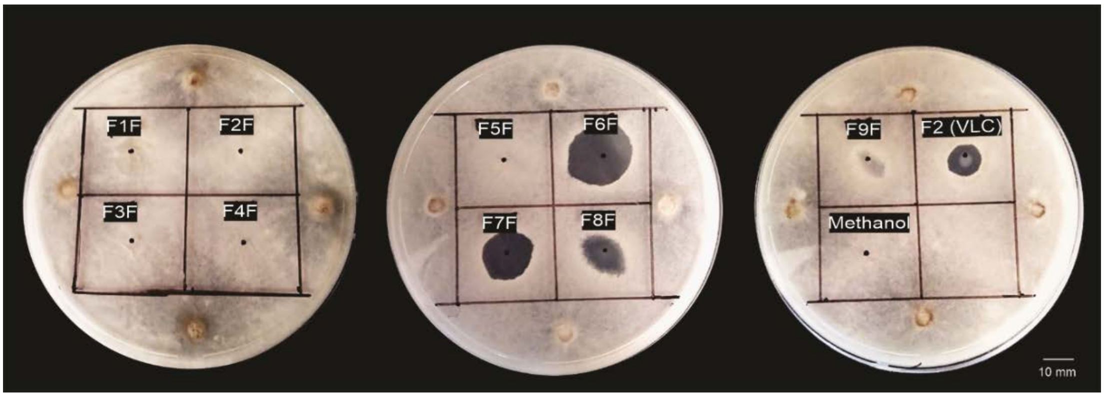

Legend. Spot-on-Lawn test of fractions obtained by FLC carried out with F2 VLC. Inhibition halos were produced in the Spot-on-Lawn assay using 250 µg of fractions F1F-F9F diluted in methanol. Among the nine obtained fractions, F6F, F7F, and F8F exhibited antifungal activity against *S. sclerotiorum*, with inhibition halos measuring 26.25 ± 1.8, 23 ± 1.0, and 15 ± 0.5 mm, respectively.

Figure S2

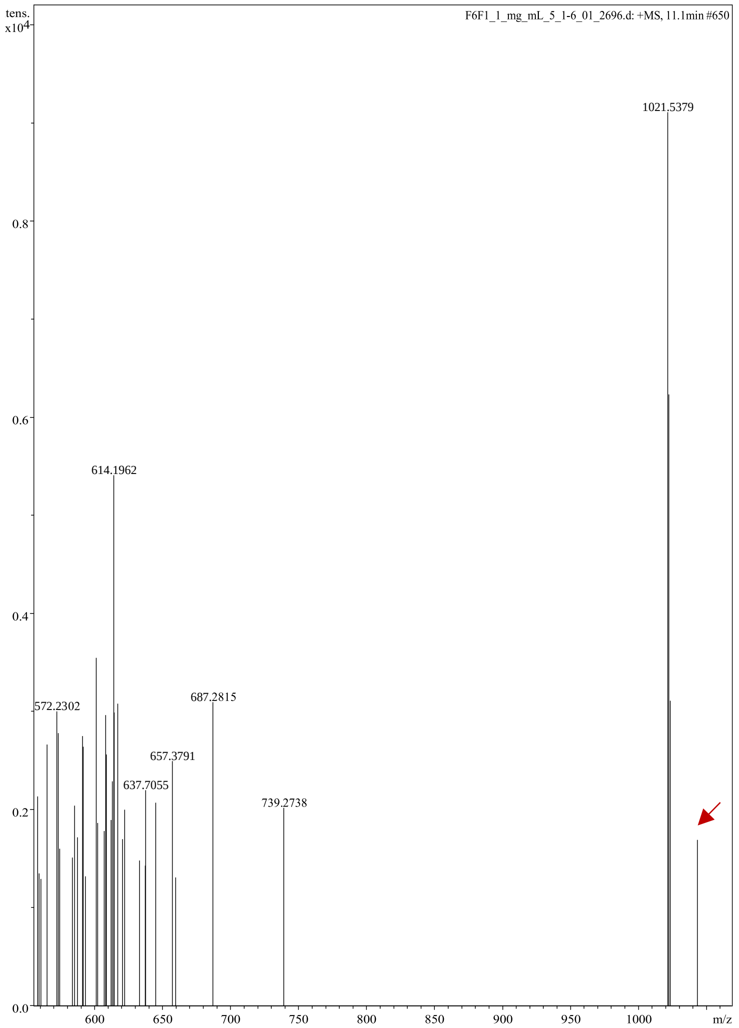

Legend. HR-ESI-MS spectrum of compound **A**. The peak at m/z 1021.5379 corresponds to the $[M+H]^+$ of component A (bacillopeptin A). The arrow at m/z 1043.4992 indicates the $[M+Na]^+$ of component A.

Figure S3

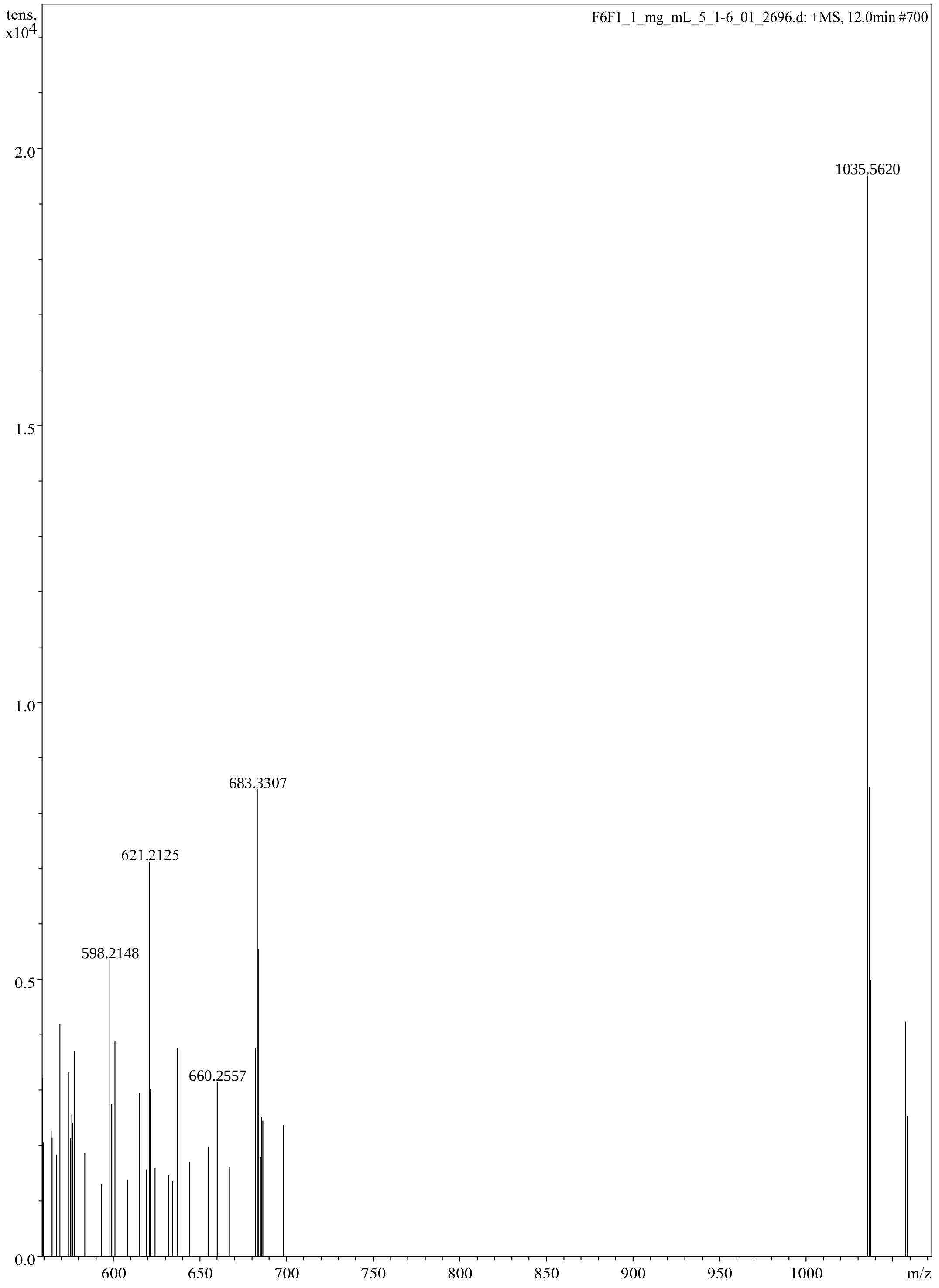

Legend. HR-ESI-MS spectrum of compound **B**. The peak at m/z 1035.5620 corresponds to the $[M+H]^+$ of component B (bacillopeptin B). The arrow at m/z 1057.5418 indicates the $[M+Na]^+$ of component B.

Figure S4

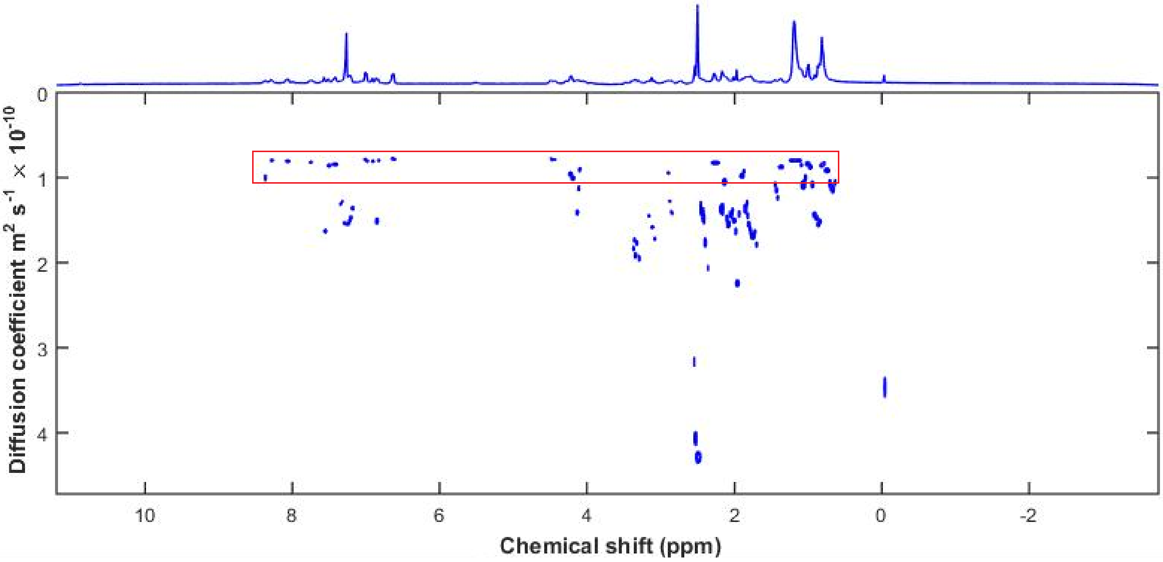

Legend. DOSY spectrum of compound **A** and **B**, showing in the red rectangle the signals that are in the range 0.8.10-10 m^2 s^{-1} and 0.9x10-10 m^2 s^{-1}. Using DOSY, it was possible to identify the set of NMR peaks in F6F.1 that correspond to metabolites within the target molecular weight range, as determined by HRMS.

Figure S5

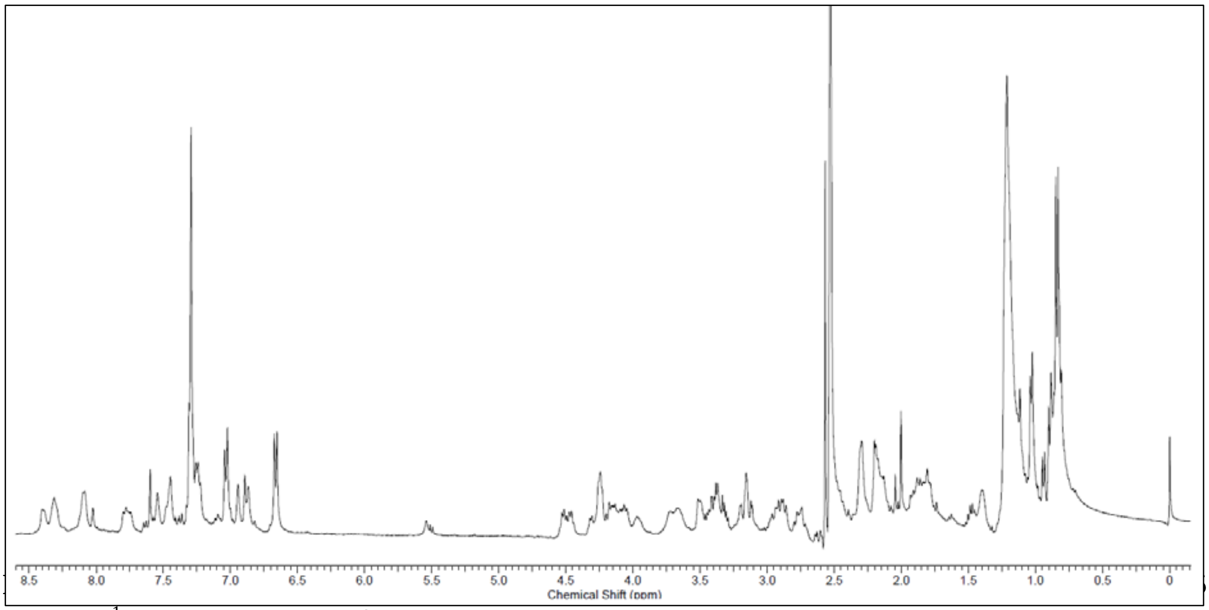

Legend. ^{1}H NMR spectrum of compound **A** and **B** (DSMO-d_6, 400.13 MHz). The signals between δ 6.84-8.29 correspond to N-bonded protons (-NH- or -NH2), and those between δ 4.49-3.74 correspond to α-amide protons, consistent with a cyclic peptide composed of seven amino acid residues. The signals between δ 1.10 and 1.37 and between δ 0.80 and 0.84 indicate the presence of a p-hydroxyphenyl group, respectively. Also, signals at δ 6.64 (2-H, J = 8.31 Hz) and 7.00 (2-H, J = 8.31 Hz) indicated the presence of the p-hydroxyphenyl group of the tyrosine residue.

Figure S6

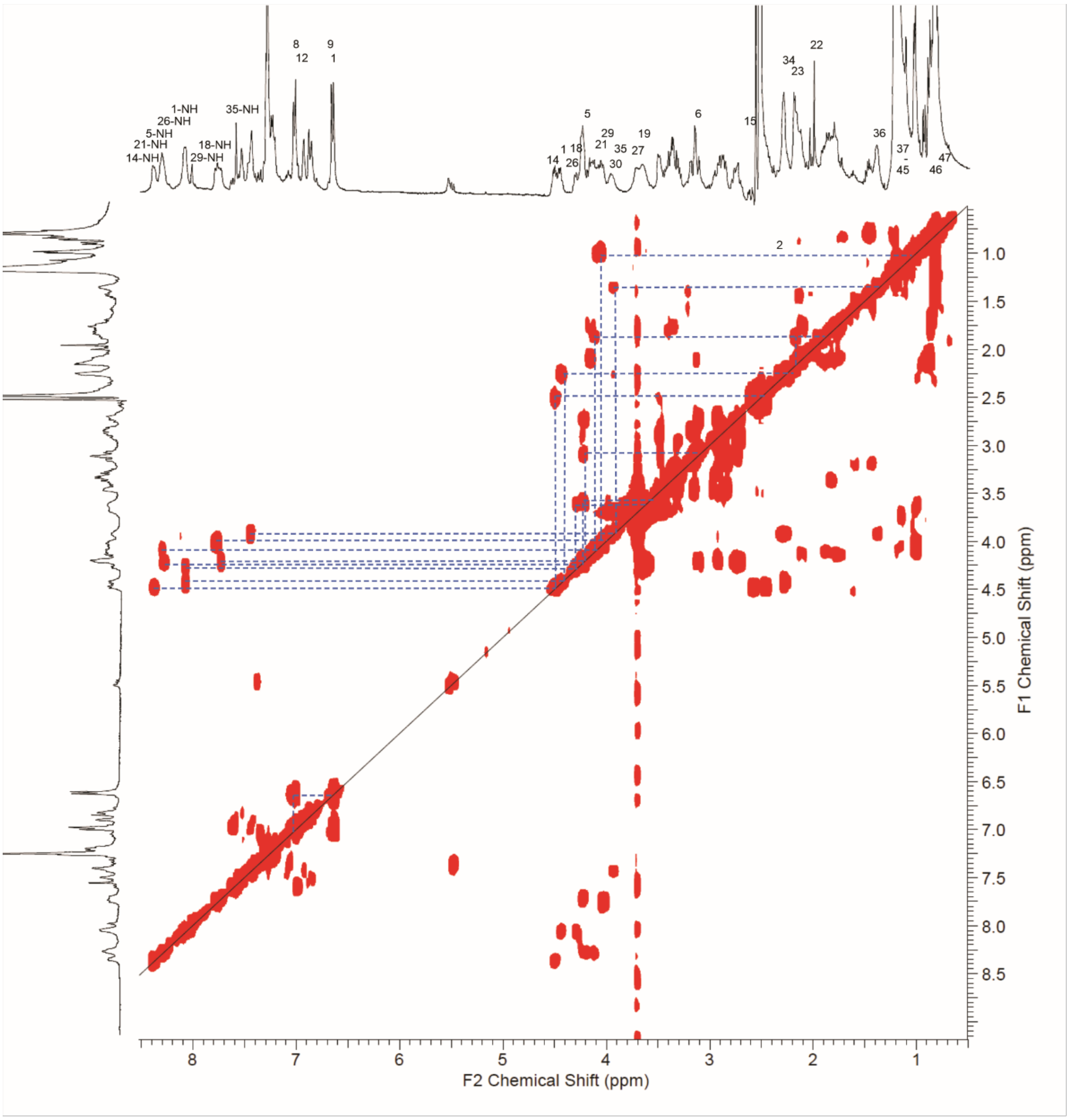

Legend. COSY spectrum of compounds **A** and **B** (DSMO-d_6). Partial ^{1}H spectrum on top. Spin systems observed for amino acid residues are shown. These spin systems helped identify the seven amino acid residues for compounds **A** and **B**.

Figure S7

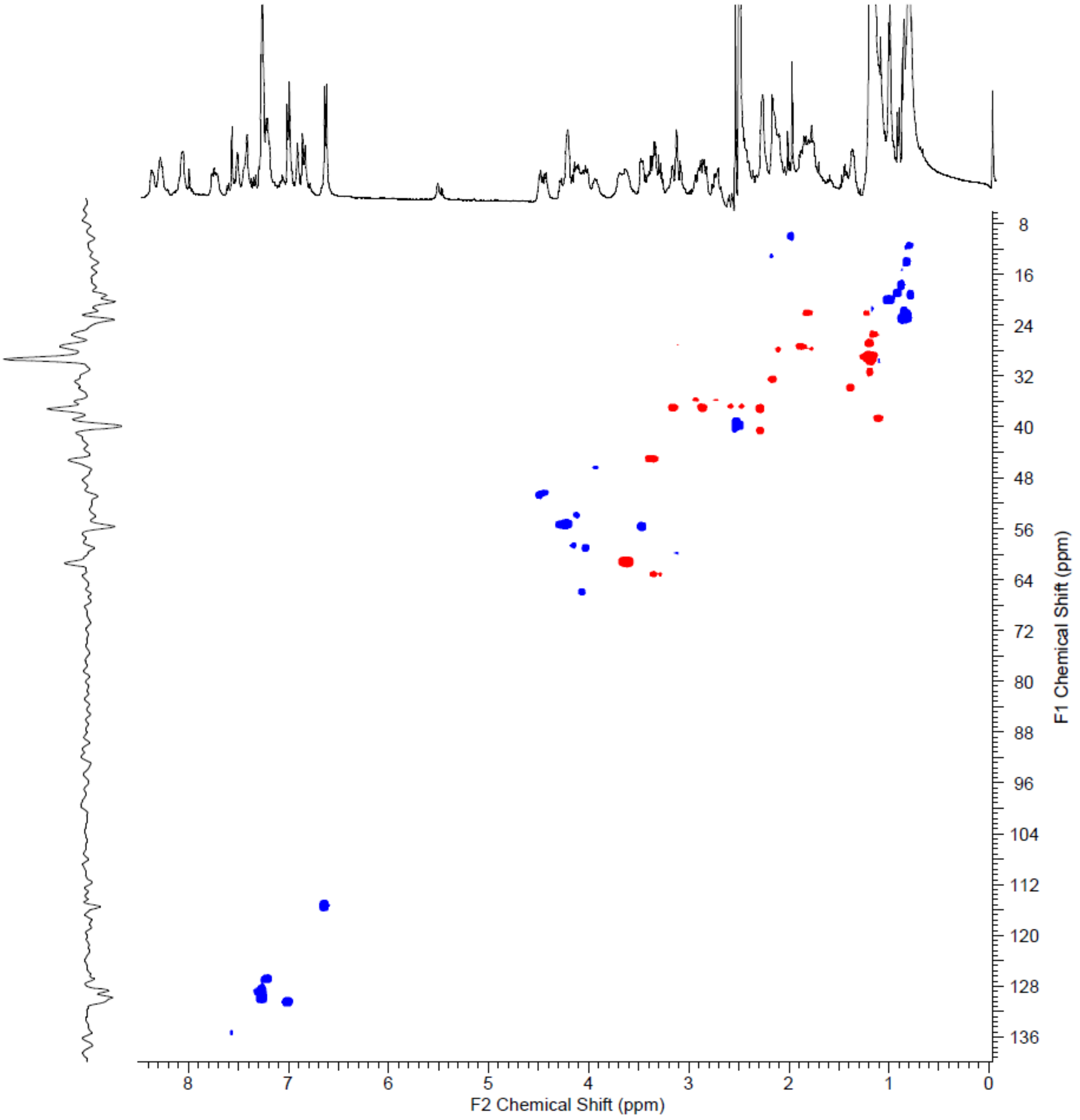

Legend. HSQC spectrum of compounds **A** and **B** (DSMO-d_6). ^{1}H spectrum on top. This experiment is used to identify protons that are one carbon bond away. It was possible to visualize the connections between H31 → C31 (1,00→ 20,7); H41 → C41 (1,19→ 29,3); H46/47 → C46/47 (0,81→ 22,4); H5 → C5 (4,23→ 55,5); H18/12 → C8/12 (7,00 → 130,5) and H9/11 → C9/11 (6,62→ 115,4).

Figure S8

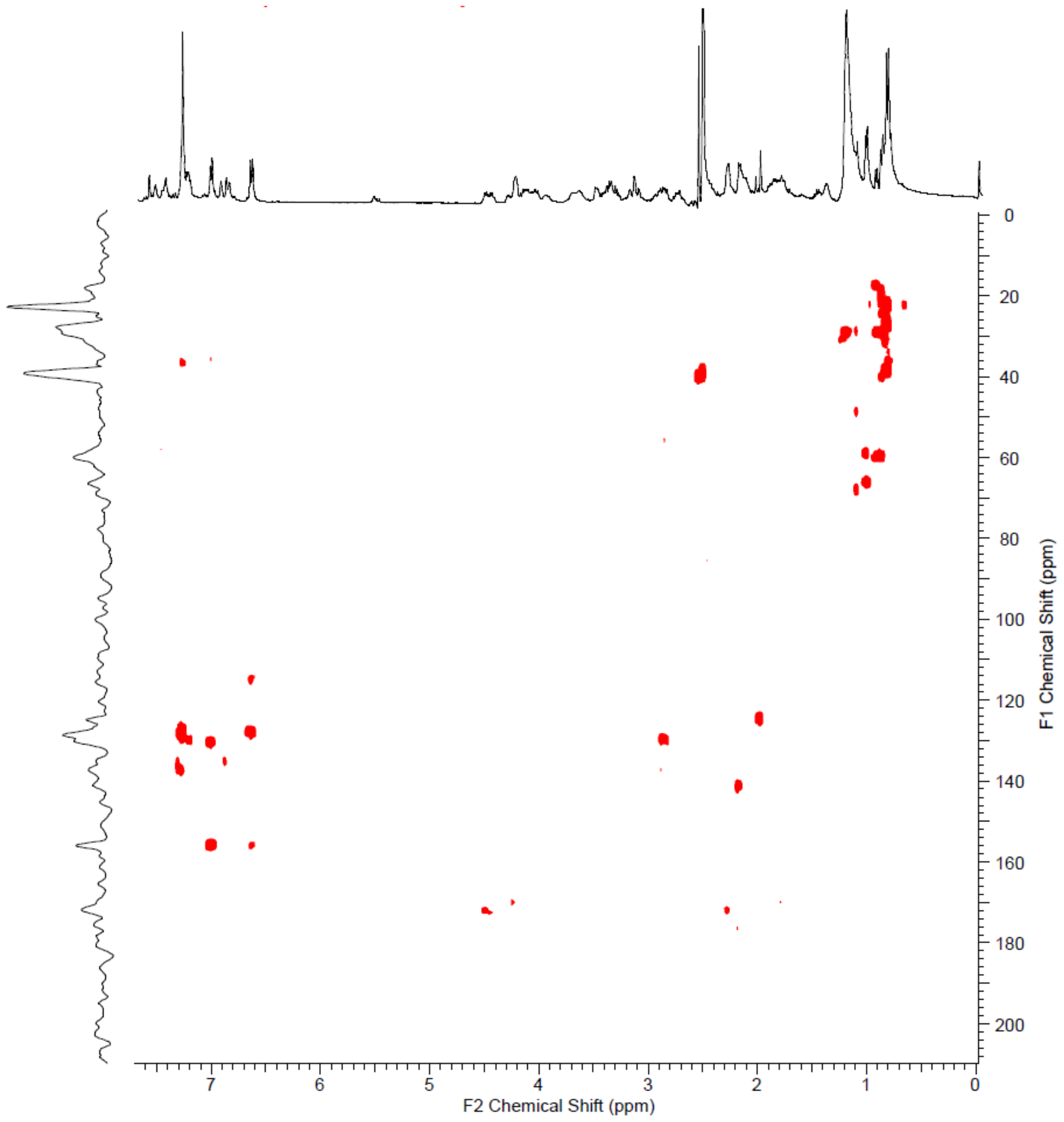

Legend. HMBC spectrum of compounds **A** and **B** (DSMO-d_6). ^{1}H spectrum on top. This experiment is used to investigate correlations between carbons and protons for two- or three-bond systems, sometimes in conjugated systems, and for four-bond systems. It was possible to visualize the connections between H08/12 → C6 (7,00 → 36,2); H18 → C19 (7,46 → 58,2); H09/11 → C7 (6,63 → 128,2); H08/12 → C10 (7,00 → 155,9); H09/11 → C10 (6,63 → 156,25); H09 → C11 (6,63 → 115,8); H08/12 → C8/12 (7,00 → 130,7); H01 → C3 (4,48 → 172,0); H02 → C4 (2,28 → 171,9); H14 → C16 (4,43 → 172,5); H18 → C20 (4,27 → 170,1); H02 → C4 (2,28 → 171,9); H31 → C30 (1,00 → 66,4); H31 → C29 (1,00 → 59,3) and H37 → C35 (1,10 → 48,9).

Figure S9

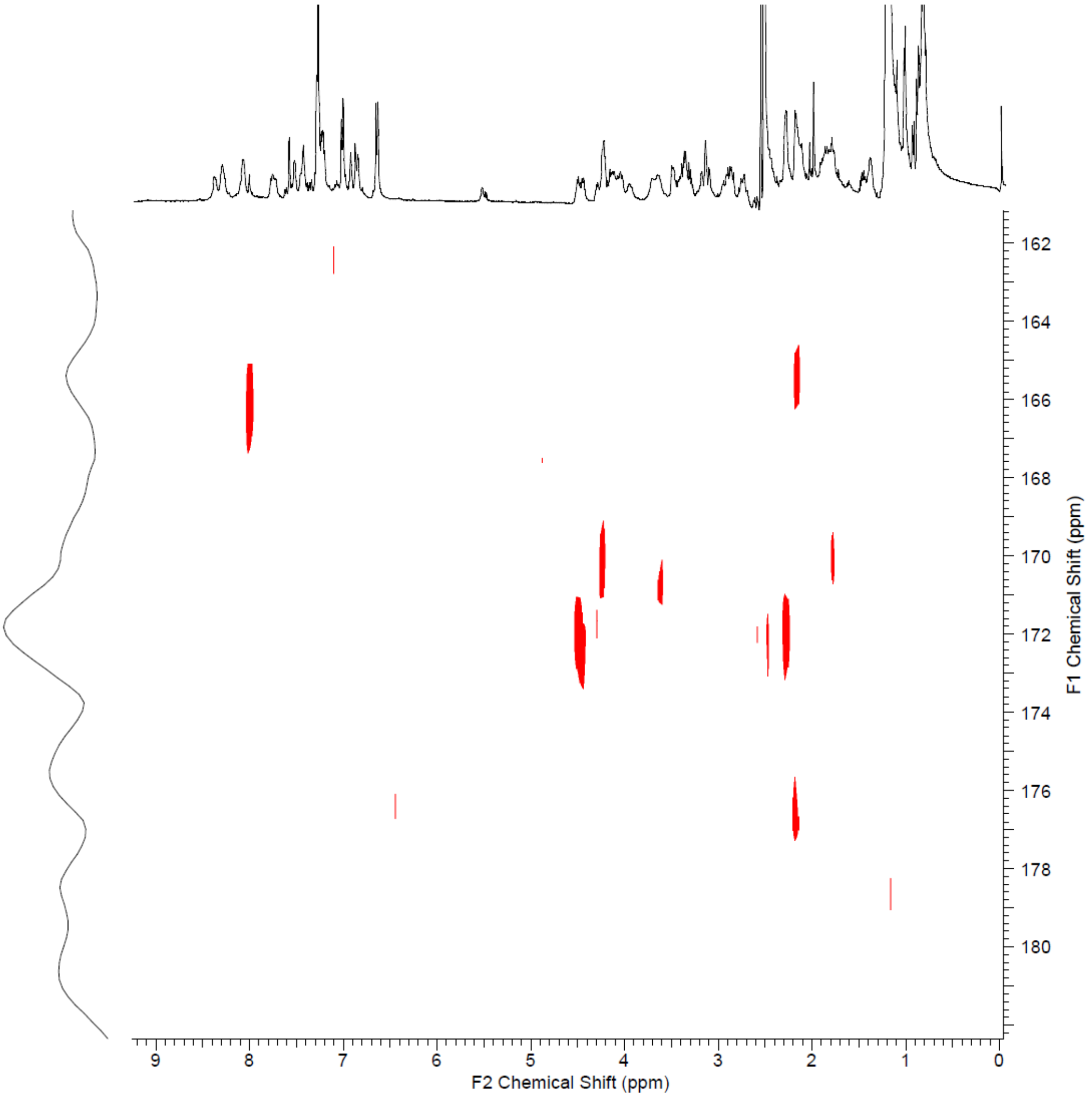

Legend. Selected region of HMBC spectrum of compounds **A** and **B** (DSMO-d_6). ^{1}H spectrum on top. In this region of the spectrum, it was possible to observe the bonds of the final lipid portion of lipopeptides: H46 → C45 (0.80 → 27.7) of the bacillopeptin A and H46 → C44 (0.80 → 38.7); H47 → C44 (0.82 → 38.7) of the bacillopeptin B.

Figure S10

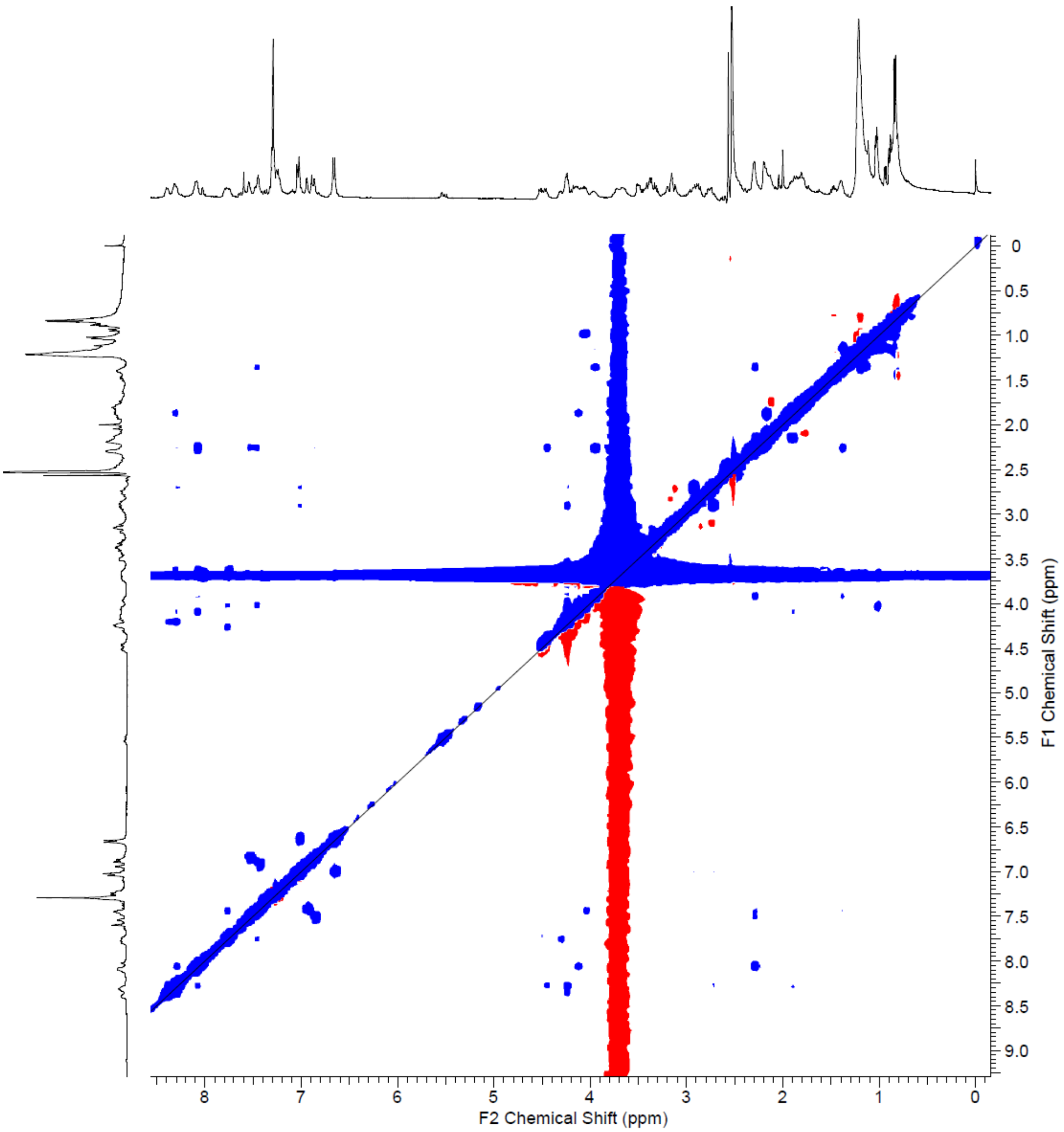

Legend. NOESY spectrum of compounds **A** and **B** (DSMO-d_6). ^{1}H spectrum on top and left side. This spectrum shows the spatial interactions between atoms. From this, it was possible to observe the correlation H01 → NH5 (4,47 ppm → 8,29 ppm).

Figure S11

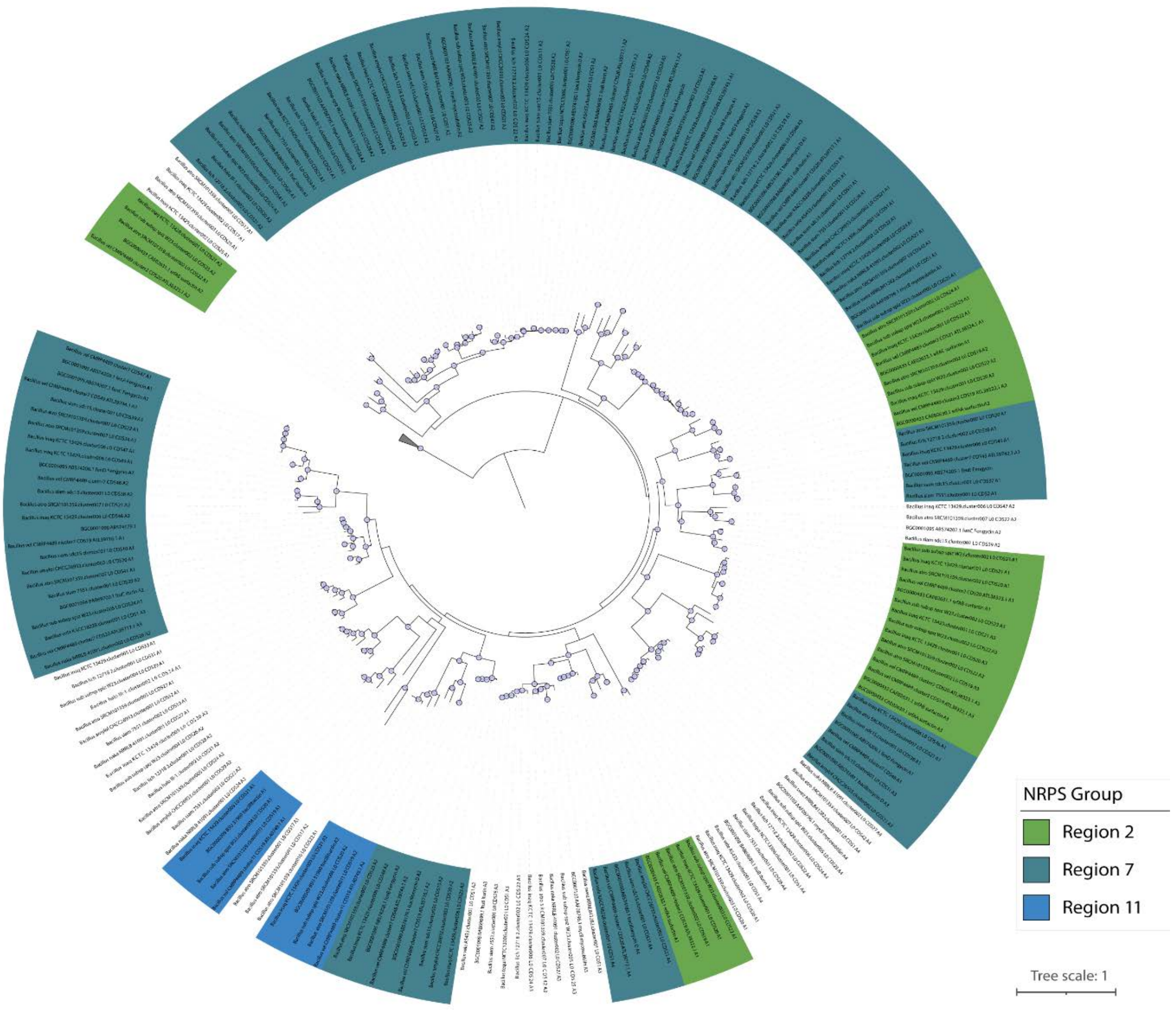

Legend. Maximum-likelihood phylogenetic tree of the adenylation domains extracted from the non-ribosomal peptide synthetase (NRPS) core enzyme of B. velezensis CMRP4489 (in bold), compared with *Bacillus* spp. from Dunlap et al. (2019) and BCG sequences deposited in the MIBiG database. The highlighted regions indicate clusters with BGC from B. velezensis CMRP4489: region 2 with surfactin; region 7 with fengycin (iturin class); and region 11 with bacillibactin. This was used to select the clades for similarity analyses, in which adenylation enzymes from *B. velezensis* CMRP4489 have been grouped.
